# Exploring structural antigens of yellow fever virus to design multi-epitope subunit vaccine candidate by utilizing an immuno-informatics approach

**DOI:** 10.1186/s43141-023-00621-7

**Published:** 2023-12-05

**Authors:** Kiran Sura, Himanshi Rohilla, Dev Kumar, Ritu Jakhar, Vaishali Ahlawat, Deepshikha Kaushik, Mehak Dangi, Anil Kumar Chhillar

**Affiliations:** 1 Centre for Bioinformatics, M.D. University, Rohtak, Haryana India; 2 Centre for Biotechnology, M.D. University, Rohtak, Haryana India

**Keywords:** Yellow fever virus, Immunoinformatics, Vaccine, MESV, Molecular docking, Molecular dynamics simulation

## Abstract

**Background:**

Yellow fever is a mosquito-borne viral hemorrhagic disease transmitted by several species of virus-infected mosquitoes endemic to tropical regions of Central and South America and Africa. Earlier in the twentieth century, mass vaccination integrated with mosquito control was implemented to eradicate the yellow fever virus. However, regular outbreaks occur in these regions which pose a threat to travelers and residents of Africa and South America. There is no specific antiviral therapy, but there can be an effective peptide-based vaccine candidate to combat infection caused by the virus. Therefore, the study aims to design a multi-epitope-based subunit vaccine (MESV) construct against the yellow fever virus to reduce the time and cost using reverse vaccinology (RV) approach.

**Methods:**

Yellow fever virus contains 10,233 nucleotides that encode for 10 proteins (C, prM, E, NS1, NS2A, NS2B, NS3, NS4A, NS4B, and NS5) including 3 structural and 7 non-structural proteins. Structural proteins—precursor membrane protein (prM) and envelope protein (E)—were taken as a target for B cell and T cell epitope screening. Further, various immunoinformatics approaches were employed to FASTA sequences of structural proteins to retrieve B cell and T cell epitopes. MESV was constructed from these epitopes based on allergenicity, antigenicity and immunogenicity, toxicity, conservancy, and population coverage followed by structure prediction. The efficacy of the MESV construct to bind with human TLR-3, TLR-4, and TLR-8 were evaluated using molecular docking and simulation studies. Finally, in-silico cloning of vaccine construct was performed withpBR322 *Escherichia coli* expression system using codon optimization.

**Results:**

Predicted epitopes evaluated and selected for MESV construction were found stable, non-allergenic, highly antigenic, and global population coverage of 68.03% according to in-silico analysis. However, this can be further tested in in-vitro and in-vivo investigations. Epitopes were sequentially merged to construct a MESV consisting of 393 amino acids using adjuvant and linkers. Molecular docking and simulation studies revealed stable and high-affinity interactions. Furthermore, in-silico immune response graphs showed effective immune response generation. Finally, higher CAI value ensured high gene expression of vaccine in the host cell.

**Conclusion:**

The designed MESV construct in the present in-silico study can be effective in generating an immune response against the yellow fever virus. Therefore, to prevent yellow fever, it can be an effective vaccine candidate. However, further downstream, in-vitro study is required.

## Background

**Fig. 1:**
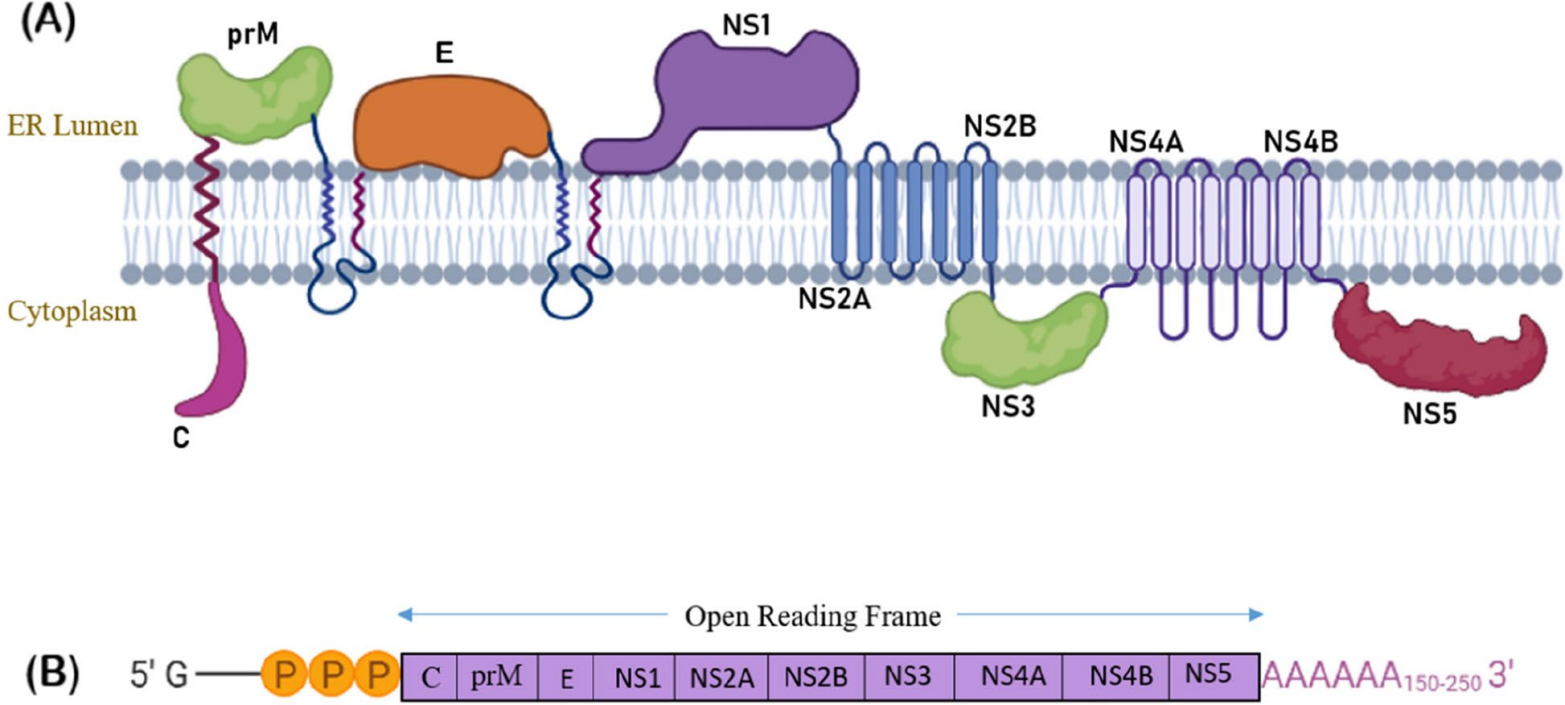
Detailed structural and non-structural proteins of yellow fever virus. **A** exhibits the localisation of these proteins on the surface of yellow fever virus, whereas **B** represents the ORF (Open Reading Frame) coding for the given structural and non-structural proteins

Yellow fever is a mosquito-borne disease transmitted via the bite of an infected Female *Aedes aegypti mosquito* [1].The *Flavivirus* genus of the *flaviviridae* family contains 70 distinct viruses, all of which are arthropod-borne, that include yellow fever virus (YFV) as well [2]. The viral disease is endemic to tropical regions of Central and South America and Africa [3].Yellow fever was first identified in Africa, and then cases were reported in Europe and North America. It contains 10,233 nucleotides that encode for 7 non-structural and 3 structural proteins (C, prM, E, NS1, NS2A, NS2B, NS3, NS4A, NS4B, and NS5) shown in Fig. 1 [4]. Earlier in the twentieth century, vaccination together with mosquito control measures was used to eliminate the yellow fever virus disease [5]. However, frequent outbreaks take place in these endemic areas due to lack of effective antiviral therapy, posing a threat to both visitors and residents of Africa and South America [6]. Recently, Kenya reported a total of 53 suspected cases of the latest yellow fever virus outbreak, including six fatalities between 12 January and 15 March 2022, affecting 11 wards of Isiolo country [7]. Consequently, yellow fever is considered as reemerging disease.

The traditional approach for designing the vaccine entails cultivating the pathogenic micro-organisms and its extraction using microbiological, biochemical, and immunological approaches to determine the components critical for immunity [8]. In this new era of vaccine research, vaccinomics term has been used to describe for designing of vaccines using reverse vaccinology approach integrated with bioinformatics as a result of the availability of massive genomic sequencing data in silico, eliminating the need to cultivate the pathogenic viruseseliminates the accidental exposure to the virus [9].

**Fig. 2:**
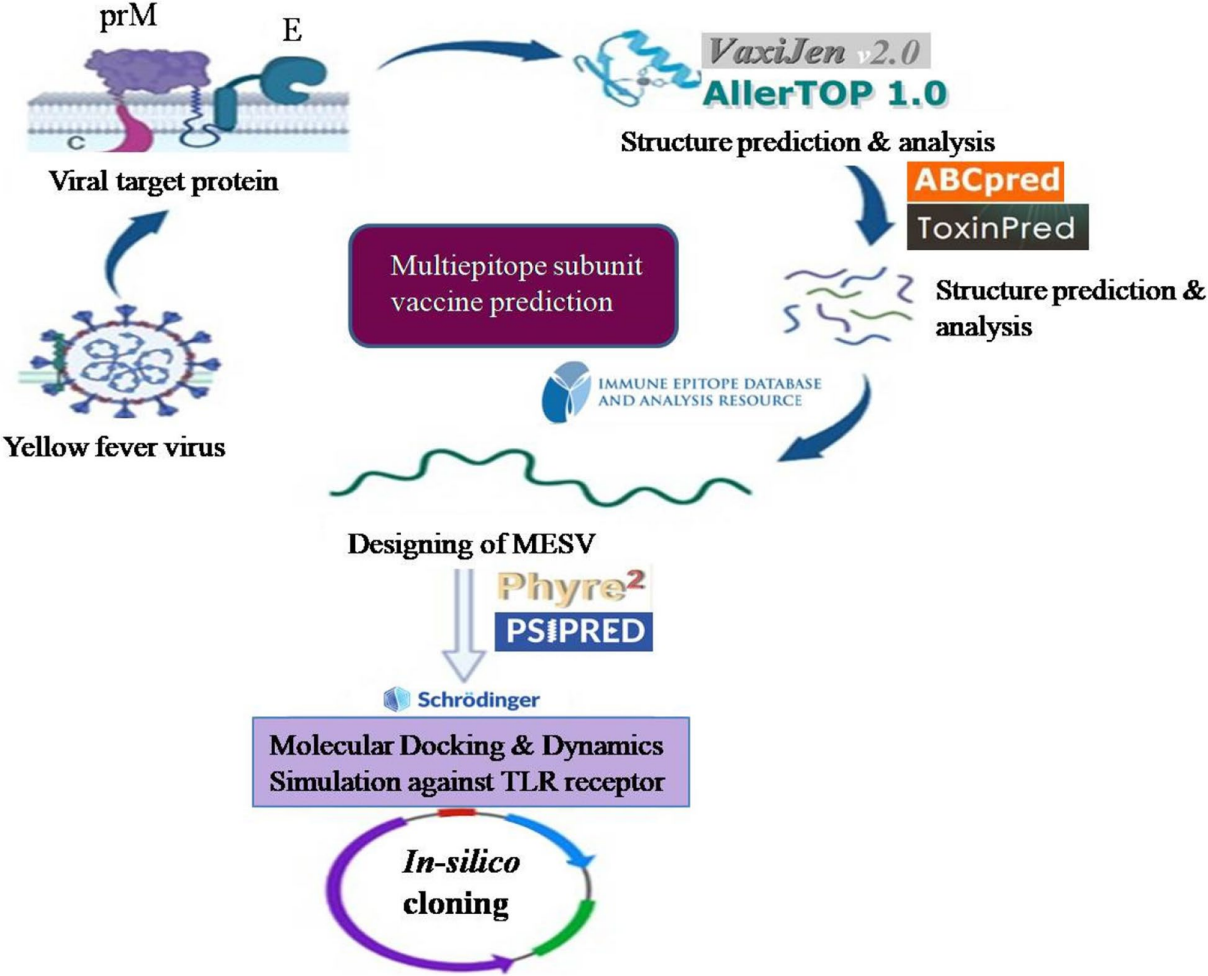
Overall schematic workflow of designing vaccine construct against structural proteins of yellow fever virus using Immunoinformatics approach

Moreover, the genome database of the National Centre of Biotechnology Information (NCBI) provides genetically mapped information and sequencing data derived from genomes of microbes and viruses. Reverse vaccinology is a strategy for predicting vaccine targets from microbe genome sequences by identifying the proteins that are exposed on the surface that could trigger an immunological response in the host organism [10, 11]. RV approach reduces the time and cost required for vaccine target detection and construction of potential vaccine candidate generating promising outcomes against various pathogenic organisms [12]. In this approach, using several bioinformatics tools, we primarily identified potential epitopes from structural proteins of the yellow fever virus; for B cell epitopes detection ABCpred was used, and for T cell epitopes, the IEDB consensus method was utilized followed by joining of epitopes with linkers and adjuvants to construct a multi-epitope subunit vaccine (MESV) [13, 14]. The physicochemical and structural characteristics of the MESV were validated and analyzed using a variety of in-silico methods [15]. Additionally, to evaluate the efficiency and effectiveness of the vaccine, molecular docking and dynamic simulations against human pathogenic toll-like receptors TLR-3, TLR-4, and TLR-8 were carried out [16]. The respective TLR’s have been thoroughly studied and found to play a crucial role in antiviral innate and humoral immunity generation [17, 18]. Furthermore, to validate the immunogenic potential of the developed MESV candidate, in silico immune simulation was also carried out [19]. The general schematic workflow of multi-epitope vaccine construction against the yellow fever virus using immunoinformatics approach is shown in Fig. 2.

## Materials and methods

### Sequence and structural analysis of target proteins

Yellow fever virus consists of 7 non-structural proteins (NS1, NS2A, NS2B, NS3, NS4A, NS4B, and NS5) and 3 structural proteins (C, prM, and E) [20]. These structural proteins were selected as target candidates for the screening of T and B cell epitopes and the designing of MESV. The amino acid sequences of the target structural proteins were downloaded from the UniProt database in FASTA format. Precursor membrane protein (prM), an envelope protein (E), and capsid protein (C) were tested based on their antigenicity and allergenicity using VaxiJen v2.0 and AllerTOP v2.0, respectively [21, 22]. The 2D structure of prM and envelope protein was predicted using PSIPRED server [23]. Three-dimensional (3D) structure of the target envelope protein (E) was retrieved from RCSB-PDB. Due to the non-availability of the 3D structure, the prM protein was predicted by PHYRE2 Protein Fold Recognition server using the protein homology modeling approach [24]. Chimera was used to visualize the predicted 3D models of the prM structure. To refine the predicted 3D structure of prM protein, Galaxy refine server was used [25]. To validate the refined structure based on experimentally derived 3D structure of proteins, the overall quality score, or z-score, of a given structure was predicted using PROSA web server [26]. Ramachandran plots for given structural proteins were created using saves v6.0 where the PROCHECK principle was applied to predict the quality of the validated structure [27].

### B cell and T cell epitope prediction

The linear and conformational B cell epitopes help in the detection of viral infections in the immune system. Consequently, B cell epitopes play a very crucial role in designing peptide vaccines. Using an artificial neural network, the ABCpred server was employed to predict the linear B cell epitopes for the target protein sequences [28]. B cell epitope prediction was based on antigenicity, allergenicity, flexibility, hydrophilicity, surface accessibility, and linear epitope prediction. Allergenicity and antigenicity of epitopes were predicted using AllerTOP v2.0 and VaxiJen v2.0 webserver at 0.5 threshold. Flexibility, accessibility of surface, hydrophilicity analysis, and antigenicity was assessed using the Karplus and Schulz flexibility prediction tool, Emini surface accessibility prediction method, Parker hydrophobicity prediction algorithm, and Kolaskar and Tongaonkar antigenicity scale. To further analyze the discontinuous epitopes from structural proteins, DiscoTope 2.0 server provided by IEDB was used [29].

Additionally, the development of vaccines also heavily relies on T cell epitopes. Moreover, compared to wet lab experiments, it saves time and cost consumption. Web-based server IEDB based on artificial neural network (ANN) and stabilized matrix method (SMM) was utilized in predicting MHC class-1 and MHC class-2 allele-compatible epitopes [30].The IEDB-based prediction approach was used to select all the human-specific alleles for T cell epitope prediction [31]. Epitopes having a consensus score of less than two were selected for continuation and considered as good binders.

### Evaluation and analysis of predicted epitopes

Vaxijen v2.0 and AllerTOP v2.0 were utilized to evaluate the antigenicity and allergenicity of the selected epitope candidates. To predict the digestive enzymes acting on epitopes, a web-based Protein Digest server was employed. To predict the toxic or nontoxic nature of epitopes, the ToxinPred server was employed, and non-toxic epitopes were chosen for further analysis [32]. Immunogenicity of the epitopes was calculated using IEDB server. Moreover, all the selected epitopes were analyzed for their physicochemical profiling including (a) half–life, (b) hydropathy, (c) aliphatic-index, (d) isoelectric point (pI), and (e) instability index was predicted using ProtParam tool [33].

### Epitope conservancy and population coverage calculation

The degree of conservation of anticipated B cell and T cell epitopes within the protein sequences was examined using the IEDB Conservancy analysis tool. The IEDB Population Coverage tool was used to calculate the distribution and expression of HLA alleles all across the population in each subcontinent area according to the Population Coverage criteria. According to the IEDB Population Coverage tool, it showed that the selected epitopes included in the given study would cover the majority of the global population [34].

### Designing of MESV construct

To construct a multi-epitope-based vaccine, epitopes satisfying the following characteristics were shortlisted to design the MESV construct: highly antigenic, immunogenic, non-toxic, non-allergenic, significant population coverage, and overlapping epitopes. All the selected overlapped B cell and T cell epitopes were conjugated together by using GPGPG and AAY linkers to enhance the immunogenicity, whereas B cell epitopes were joined by KK linkers. An adjuvant β-defensin 45 amino acids sequence was added to the first cytotoxic T lymphocytes (MHC-1/CTL) on the N-terminal of the MESV construct sequence with the help of EAAAK linker. It functions as both an antimicrobial agent and an immunomodulator**.** Blastp was performed to evaluate the similarity and homogeneity of the designed MESV construct against Homo sapiens proteome [35, 36].

### Evaluation of designed MESV construct

#### Allergenicity and antigenicity

To evaluate the allergenicity prediction of the MESV construct, the AllerTOP v2.0 server was utilized for in-silico allergen prediction based on the primary physicochemical characteristics of proteins. Web-based server VaxiJen v2.0 was employed to predict the antigenicity of the designed MESV construct.

#### Prediction of secondary structure of MESV construct and analysis

To predict the secondary structure of MESV, PSIPRED server was employed based on a feed-forward artificial neural network. This evaluates various vaccine properties like alpha helices, degree of beta turns, random coil, and extended chain.

#### Prediction of 3D structure of MESV construct: homology modeling

3D structure of the designed vaccine was predicted using Phyre2: Protein Fold Recognition server based on the protein homology modeling approach. The FASTA sequence of the MESV construct was provided to the Phyre2 server in intensive modeling mode since the construct was having a series of epitopes and no template was available for the MESV construct. Phyre2 employs alignment of HMM resulting in increased accuracy of alignment and prediction of structure.

Chimera was used to visualize and to minimize the energy of the vaccine construct’s predicted 3D structure. Galaxy Refine web server was utilized to refine the best 3D structure of the designed MESV construct based on ERRAT and Z-score. Further, the structure was validated by Ramachandran plot analysis using RAMPAGE, ProSA-web followed by ERRAT server for evaluating the structural accuracy.

Linear and conformational epitopes of MESV were predicted using ABCPred and Ellipro server, respectively. It predicts epitopes by protein shape, neighbor residue clustering, and estimating protrusion index (pI).

### Molecular docking of MESV with human TLR-3 (1ziw), TLR-4 (3fxi), TLR-8 (3wn4) using Schrodinger Maestro

The PDB structure of TLR receptors was retrieved from RCSB PDB database. Preprocessing of MESV construct and TLR receptors was performed in the Maestro, Protein Preparation Wizard, which includes optimization and minimization of complexes. Information on binding site residues of the target proteins was retrieved by CastP server before performing the docking. The active site of protein was then visualized using Discovery Studio.

Protein–protein molecular docking was performed using Schrodinger Maestro to determine the binding affinities and interaction patterns between the TLR receptors (TLR3:1ziw, TLR4:3fxi, TLR8:3wn4) and designed multi-epitope vaccine construct. Specify the TLR’s binding site residues constraints derived from CastP in Protein–Protein Docking Wizard. The vaccine-TLR complex having the lowest docking energy score and best interacting conformation within the binding groove was selected. Further, various molecular interactions like hydrophobic, H-bond, and vdW in the docked complex were visualized through BIOVIA Discovery Studio Visualizer Software.

### Molecular dynamics simulation

Molecular dynamics simulation of vaccine/TLR complex having the best docking energy score was performed for 100 ns using Desmond, a package of Schrodinger. Molecular Docking studies provide the basis for the prediction of MESV/vaccine binding state in a static condition. Since docking is a static representation of the vaccine’s binding pose in the active site of TLR’s receptor. MD simulation tends to calculate the movement of an atom over time by integrating Newton’s classical equation of motion. MD Simulations were carried out to predict the MESV binding status with TLR receptors in the physiological environment. The simulation was run using the OPLS 2005 force field, and the TIP3P (Transferable Intermolecular Interaction Potential 3 Points) solvent model with an orthorhombic box was selected. The counter ions were added to the solvent model to make it neutral. Further, 0.15 M salt (NaCl), 300 K temperature, and 1 atm pressure were selected to mimic the physiological conditions to perform the simulations. The trajectories were saved after every 10 ps for analysis, and the simulation’s stability was assessed by computing the root mean square deviation (RMSD) of the vaccine and protein over time.

### In-silico immune simulation using C-ImmSim

C-Immsim webserver was used to carry out the immune simulations of the final designed vaccine construct to determine the cellular and humoral immune response and assess the immunogenicity. The server is based on machine learning derived position-specific scoring matrices (PSSM) for predicting immunological interactions. Immune simulations were performed with default parameters with a simulation volume of 10 for 100 Simulation steps.

### Codon optimization and in-silico cloning in Escherichia coli system

To determine the potential expression of the designed multi-epitope vaccine construct, codon optimization followed by in-silico cloning was performed using the Mendelgen tool. The vaccine sequence was reverse-transcribed into cDNA and optimized using the JCat codon adaptation tool to improve the translation efficiency of the foreign gene inserted into the host. The cDNA sequence derived from the vaccine construct was cloned inside the pBR322 expression vector of *E*. *coli* in-silico.

## Results and discussion

### Retrieval of sequence and structure analysis of target protein

#### Antigenicity and allergenicity

Target structural proteins were predicted for their antigenicity and allergenicity and found to be non-allergenic and highly antigenic. Precursor membrane protein (prM) and envelope protein having the antigenic values 0.69 and 0.56, respectively were selected, whereas capsid protein was found to be non-antigenic in nature.

#### Secondary structure prediction of prM and envelope (E) protein

**Fig. 3:**
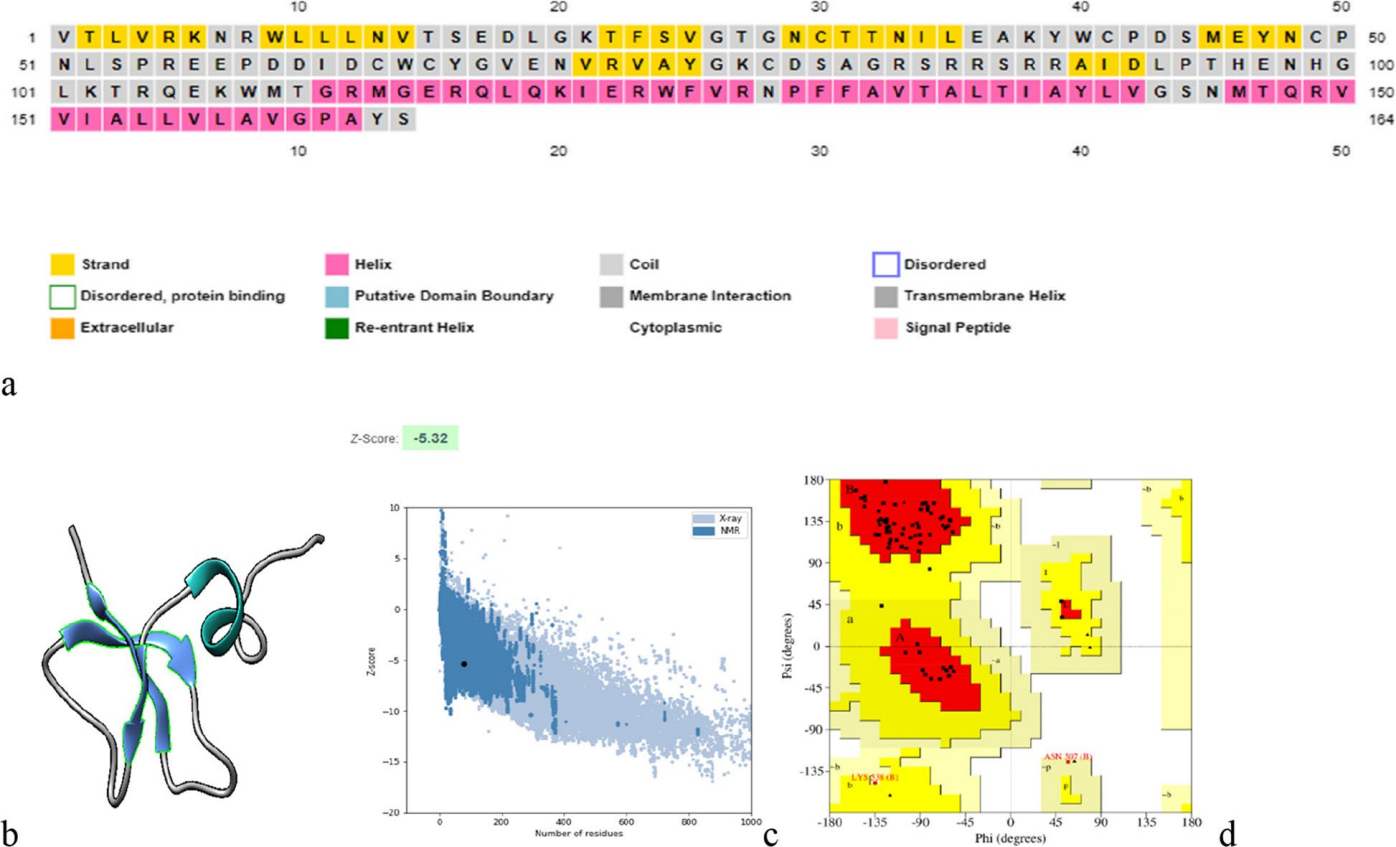
**a** The secondary structure prediction results of prM protein using PSIPRED α-helix (pink), beta strands (yellow), and random coil (gray). **b** 3D structure of prM protein visualized by chimera (alpha helix: green; beta strands: blue; coil:gray). **c** z-score (− 5.32) of the prM Protein using PROSA web. **d** Ramachandran plot of the refined structure showed 94.2%, 2.7%, and 02.9% residues in favored, allowed and 0.00% disallowed region. Using Saves v6.0, the helical, sheet, and loop regions in this structure are represented by the colors red, yellow, and green, respectively

**Fig. 4:**
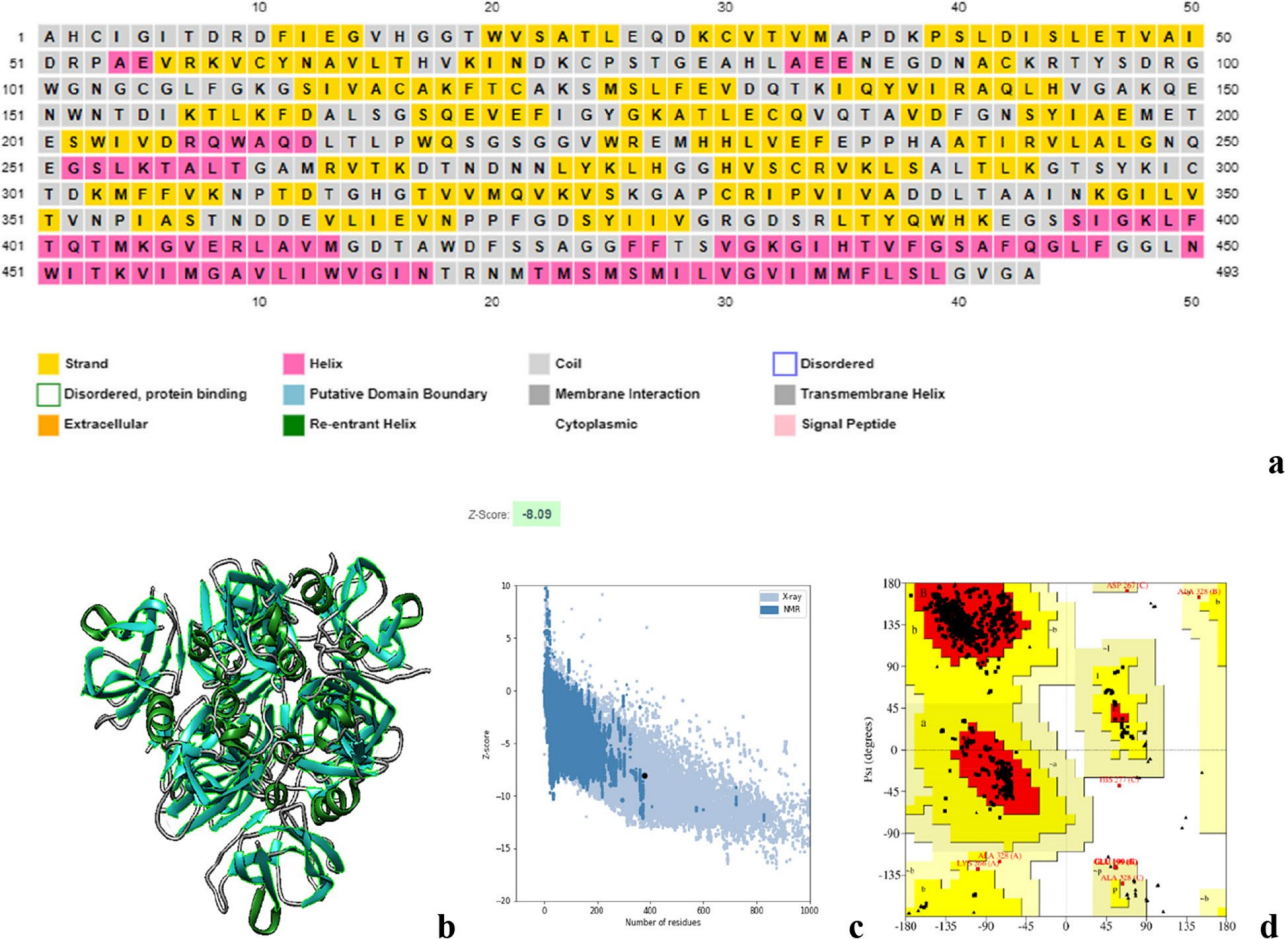
**a** The E protein consists of α-helix (pink), beta strands (yellow), and random coil (gray). **b** 3D structure of envelope protein visualized by chimera (alpha helix: green, coil: gray, beta strands: blue). **c** The z-score of the E protein (− 8.09). **d** The Ramachandran plot of the refined structure showed 93.4%, 5.7%, and 0.0% residues in favored, allowed, and disallowed region. In this structure, the helical, sheet, and loop regions are represented by the colors red, yellow, and green, respectively

The secondary structure of prM (Fig. 3) and E protein (Fig. 4) was predicted using PSIPRED and visualized by Chimera. The Z-score of prM and E protein was calculated using PROSA web. Ramachandran plot analysis showed the residues present in allowed and disallowed regions.

#### Three-dimensional structure analysis

For structural analysis, the 3D structure of envelope protein was retrieved from RCSB PDB having PDB ID: 6iw1. ERRAT score of envelope protein was found to be 87.96% and 93.4% of residues and are present in the most favored region, 5.7% present in the additional allowed region, and 0.1% in the disallowed region. However, the 3D structure of prM protein was predicted using PHYRE2 Protein Fold Recognition server. Galaxy refine server was further used to refine the predicted model of prM protein. It provided 5 models and we selected the best model based on the overall quality score and Ramachandran plot analysis. For prM, we selected Model 4 having an ERRAT score of 100 and 94.2% residues present in the favored region, 2.9% in the allowed region, and 0.0% in the disallowed region. Visualization of the model was done by Chimera.

### B cell epitopes prediction using IEDB

**Fig. 5:**
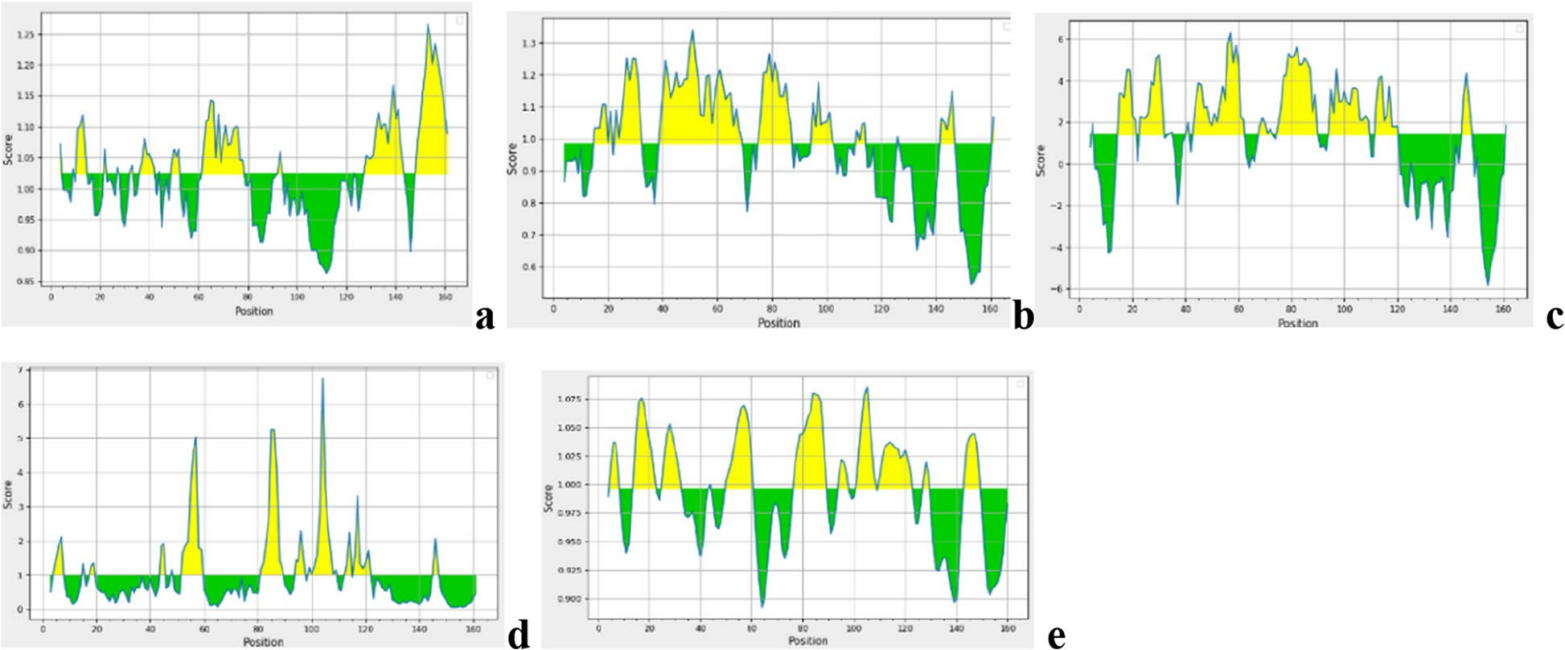
**a** Predicted antigenic determinants of prM protein using Kolaskar and Tongaonkar antigenicity scale in IEDB in which yellow peak corresponds to highly antigenic epitopes. **b** Beta turns analysis of prM protein using Chou and Fasman Beta turn prediction. **c** Parker hydrophilicity employed for hydrophilicity prediction of prM protein. **d** Represents the Emini surface accessibility prediction results of prM protein where yellow peak corresponds to the surface accessible residues Emini surface accessibility scale. **e** Flexibility analysis of prM protein using Karplus and Schulz flexibility scale where yellow peak nominates the flexible epitopes

**Fig. 6:**
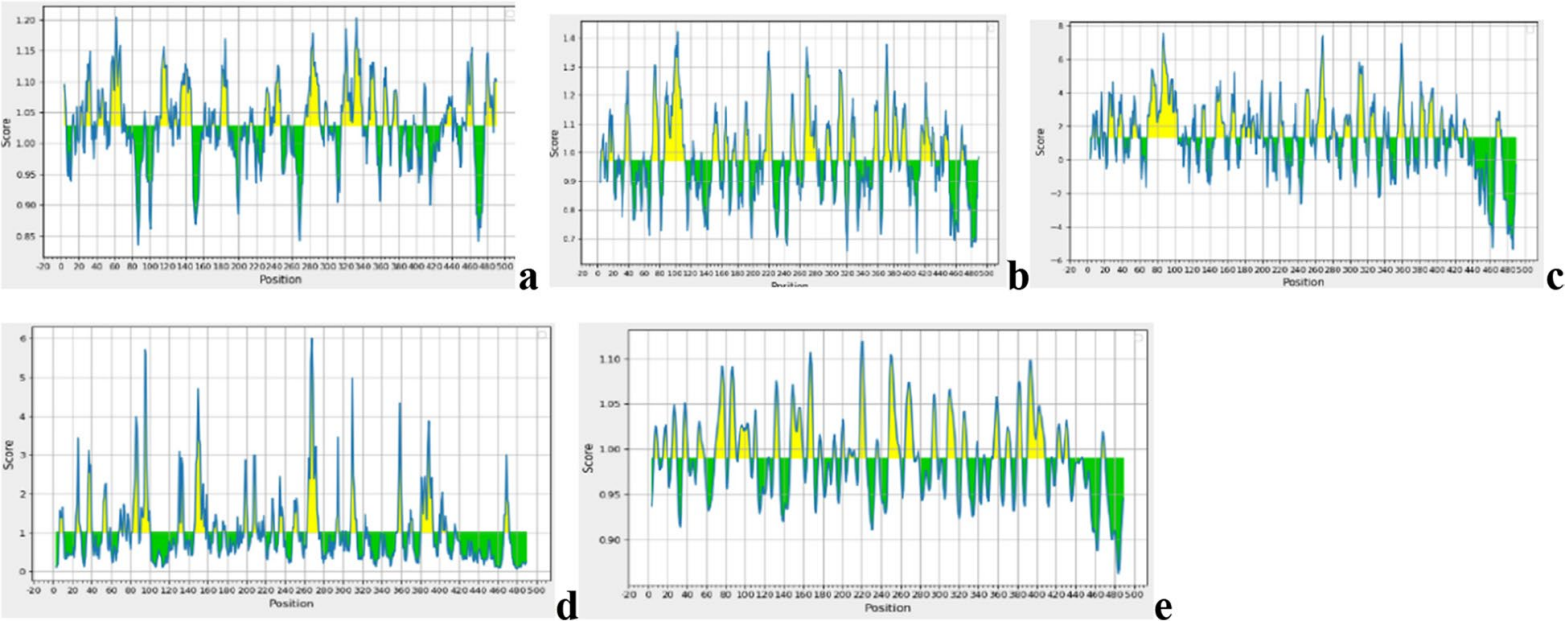
**a** Predicted antigenic determinants of E protein where yellow peak results in the highly antigenic residues using Kolaskar and Tongaonkar antigenicity scale. **b** Beta turns analysis in E protein using Chou and Fasman Beta turn prediction. **c** Hydrophilicity prediction of E protein using Parker hydrophilicity. **d** Surface accessibility analysis of E protein in which yellow peak represents highly antigenic residues using Emini surface accessibility scale. **e** Flexibility analysis of E protein in which yellow peak represents flexible residues using Karplus and Schulz flexibility scale

B cells contribute significantly to generating humoral immune responses through antibody secretion. A total of 15 linear epitopes of prM and envelope protein was predicted using the ABCpred server tabulated in Table 1. Among these selected linear epitopes, “HCIGITDRDFIEGVHG” of envelope protein has the highest antigenicity value of 1.5. Factors like antigenicity, flexibility, beta-turn analysis, surface accessibility, and hydrophobicity were the selected criteria in the server to identify the B cell epitopes. Kolaskar and Tongaonkar antigenicity scale, Chou and Fasman beta turn prediction, Karplus and Schulz flexibility prediction tool, Emini surface accessibility prediction method, and Parker hydrophilicity prediction algorithms were used to perform antigenicity, beta-turn analysis, flexibility, accessibility of surface, and hydrophilicity for prM and E proteins are shown in Figs. 5 and 6 respectively. DiscoTope 2.0 server was utilized to forecast the discontinuous B cell epitopes to improve the variety and specificity of B cell epitopes. 3D structure of prM and envelope protein were considered to forecast discontinuous B cell epitopes with 90% specificity, − 3700 thresholds, and 22.000 Å propensity score radius. A total of 36 discontinuous epitopes were predicted, 1 of prM protein and 35 of E protein.

**Table 1:** Selected B cell epitopes and their features

Epitope	Antigenic/non-antigenic	Hydrophobic/hydrophilic	Surface accessible/not surface accessible	Flexible/Not flexible	Beta turn
HCIGITDRDFIEGVHG (287–302)	Antigenic	Hydrophobic	Surface accessible	Flexible	High beta turn
NCPNLSPREEPDDI (169–182)	Antigenic	Hydrophobic	Surface accessible	Flexible	High beta turn
KVCYNAVLTHVKINDK (343–354)	Antigenic	Hydrophobic	Surface accessible	Flexible	High beta turn
AVMGDTAWDFSSAGGF (696–711)	Antigenic	Hydrophobic	Surface accessible	Flexible	High beta turn
VLIWVGINTRNMTMSM (745–760)	Antigenic	Hydrophobic	Surface accessible	Flexible	High beta turn

### T cell epitope prediction using IEDB

For the prediction of T cell epitopes, an IEDB webserver was utilized to predict the epitopes from the target structural protein sequence that bound to multiple alleles. Non-allergenic, highly antigenic, and epitopes having high population coverage and 100% conserved sequence were selected followed by allergenicity and antigenicity evaluation. T cell epitopes that follow these criteria are considered, 5 MHC-1 (3 of prM protein and 2 of E protein) and 9 MHC-2 (E protein) were shortlisted. Epitope-digesting enzymes were estimated using the Protein Digest server. Epitopes that can be digested by several enzymes are unstable. However, fewer enzyme-digested epitopes are extremely stable and are preferred as vaccine candidates.

### Evaluation and selection of T cell and B cell epitopes for designing MESV

**Fig. 7:**
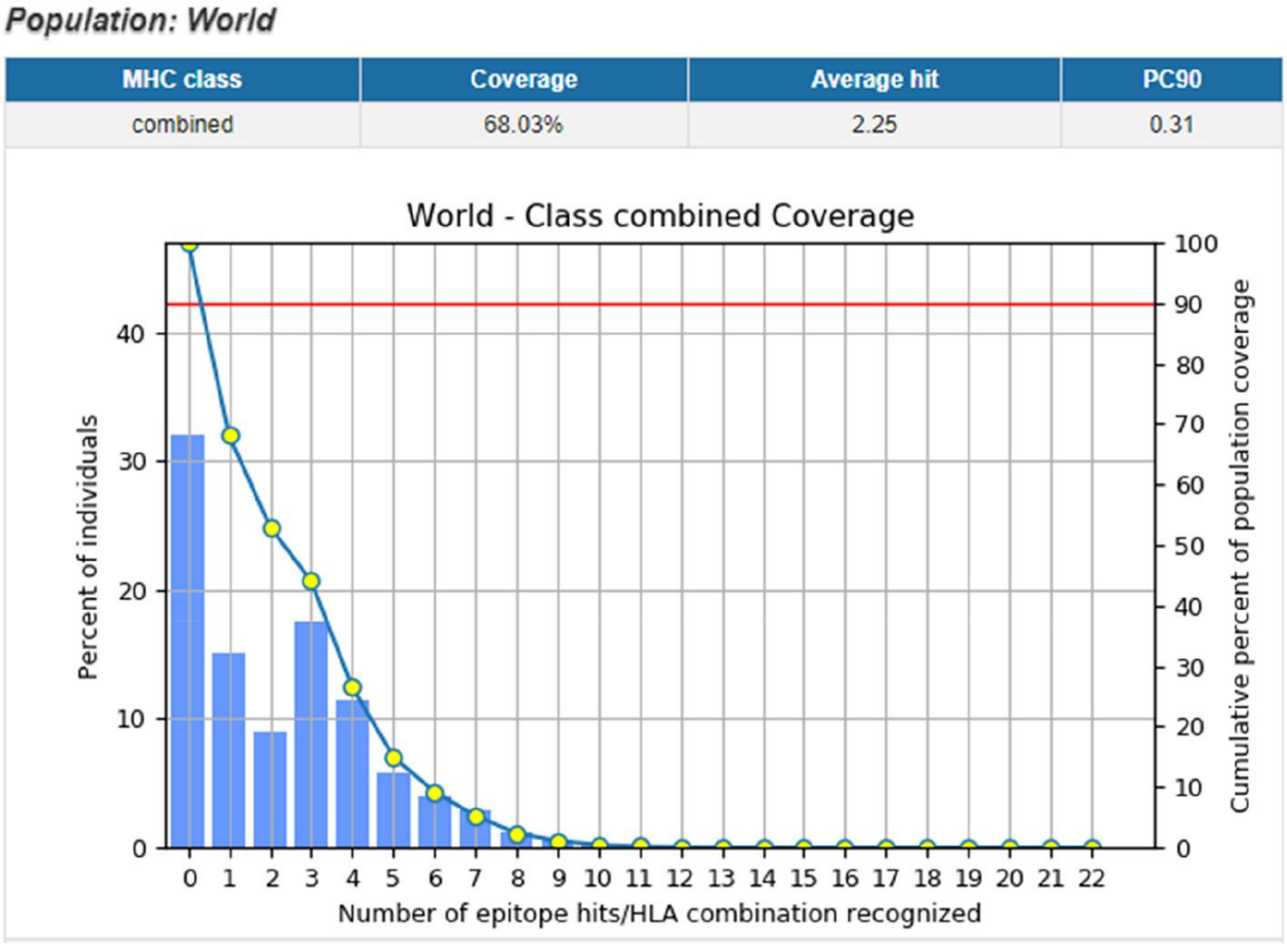
World population coverage graph

Five CTL epitopes (MHC class-1), nine HTL (MHC class-2) epitopes, and five B cell epitopes having high global population coverage were employed for designing MESV (Table 2). Results showed that the predicted epitopes having 68.03%of world population coverage that are strongly affected by yellow fever (Fig. 7).

**Table 2:** Selected MHC class-I allele, MHC class-II, and B cell binding peptides with their antigenicity scores and immunogenicity score to construct MESV

Protein	Peptide	Allele	Antigenicity	Immunogenicity
MHC class-1 prM	YGVENVRVAY (187–196)	HLA-A*29:02 HLA-A*30:02 HLA-B*15:01 HLA-B*35:01 HLA-B*46:01 HLA-C*12:03	1.5	0.25
prM	FAVTALTIAY (252–261)	HLA-A*01:01 HLA-A*26:01 HLA-A*29:02 HLA-A*80:01 HLA-B*15:01 HLA-B*35:01 HLA-B*46:01 HLA-B*53:01 HLA-B*58:01 HLA-C*12:03	1.2	0.25
prM	LLVLAVGPAY (275–284)	HLA-A*26:01 HLA-A*29:02 HLA-A*30:02 HLA-B*15:01 HLA-B*15:02 HLA-B*35:01	0.7	0.12
E	GSQEVEFIGY (451–460)	HLA-A*01:01 HLA-A*30:02	1.1	0.44
E	QTAVDFGNSY (470–470)	HLA-A*01:01 HLA-A*25:01 HLA-A*26:01 HLA-A*30:02 HLA-B*15:01	1.1	0.11
MHC class-2 (E protein)	QTKIQYVIRAQLHVG (417–431)	HLA-DRB1*03:06 HLA-DRB1*08:01 HLA-DRB1*08:13 HLA-DRB1*08:17 HLA-DRB1*11:04 HLA-DRB1*11:20 HLA-DRB1*11:28 HLA-DRB1*13:05 HLA-DRB1*15:02	0.9	0.13
E	TKIQYVIRAQLHVGA (418–432)	HLA-DRB1*03:06 HLA-DRB1*04:21 HLA-DRB1*07:01 HLA-DRB1*07:03 HLA-DRB1*08:13 HLA-DRB1*08:17 HLA-DRB1*11:20 HLA-DRB1*11:28 HLA-DRB1*13:05	0.9	0.14
E	PPHAATIRVLALGNQ (521–535)	HLA-DRB1*08:06 HLA-DRB1*11:06 HLA-DRB1*13:07 HLA-DRB1*13:11	0.8	0.38
E	PHAATIRVLALGNQE (522–536)	HLA-DRB1*11:04 HLA-DRB1*11:06 HLA-DRB1*13:07 HLA-DRB1*13:11	0.8	0.28
E	AATIRVLALGNQEGS (524–538)	HLADRB1*11:04 HLA-DRB1*11:06 HLA-DRB1*13:07 HLA-DRB1*13:11	0.8	0.9
E	ATIRVLALGNQEGSL (525–539)	HLA-DRB1*11:06 HLA-DRB1*11:14 HLA-DRB1*13:07 HLA-DRB1*13:11	0.8	0.9
E	GAVLIWVGINTRNMT (743–757)	HLA-DRB1*03:09 HLA-DRB1*04:08 HLA-DRB1*08:17 HLA-DRB1*11:02 HLA-DRB1*11:04 HLA-DRB1*11:28 HLA-DRB1*13:05 HLA-DRB1*13:23 HLA-DRB1*15:02	1	0.52
E	AVLIWVGINTRNMTM (744–758)	HLA-DRB1*04:08 HLA-DRB1*04:21 HLA-DRB1*08:06 HLA-DRB1*11:02 HLA-DRB1*11:14 HLA-DRB1*11:28 HLA-DRB1*13:05 HLA-DRB1*13:23 HLA-DRB1*15:02 HLA-DRB5*01:05	1.1	0.49
E	VLIWVGINTRNMTMS (745–759)	HLA-DRB1*04:08 HLA-DRB1*04:21 HLA-DRB1*08:06 HLA-DRB1*08:17 HLA-DRB1*11:02 HLA-DRB1*11:14 HLA-DRB1*11:28 HLA-DRB1*13:05 HLA-DRB1*13:23 HLA-DRB5*01:01	1.2	0.30
B cell	HCIGITDRDFIEGVHG (287–302)	–	1.2	0.76
	NCPNLSPREEPDDI (169–182)	–	1.4	0.07
	KVCYNAVLTHVKINDK (343–354)	–	1.1	0.07
	AVMGDTAWDFSSAGGF (696–711)	–	1.0	0.18
	VLIWVGINTRNMTMSM(745–760)	–	1.2	0.16

### Designing MESV

**Fig. 8:**
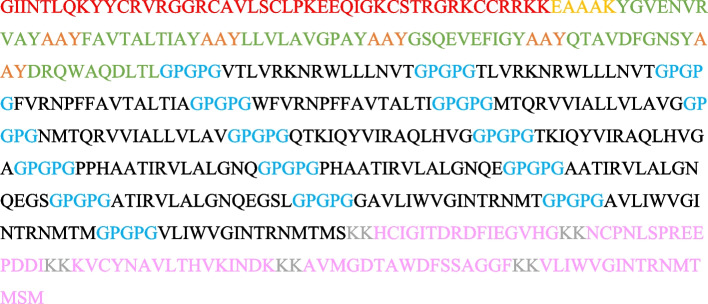
MESV construct sequence shows an adjuvant sequence in red color, EAAAK linker sequence is in gold color, AAY linker is in orange color, GPGPG linkers highlighted with blue color, KK linkers are highlighted with gray color. MHC-1 were in green blue, MHC-2 were in black, and B cell epitopes were in purple

All chosen epitopes were used to create the MESV. By using EAAAK linker, an adjuvant β-defensin was ligated to MHC-1 epitopes, each MHC-1 epitope is connected to each other with AAY linker. Epitopes were sequentially merged using AAY, GPGPG, and KK linkers. The final MESV construct consisted of 393 amino acids. MESV construct sequence showed adjuvant sequence in red color, EAAAK linker sequence is in gold color, AAY linker is in orange color, GPGPG linkers are highlighted with blue color, KK linkers are highlighted with gray color. After ligation vaccine construct obtained is shown below in Fig. 8.

### Structure modeling of MESV and analysis

**Fig. 9:**
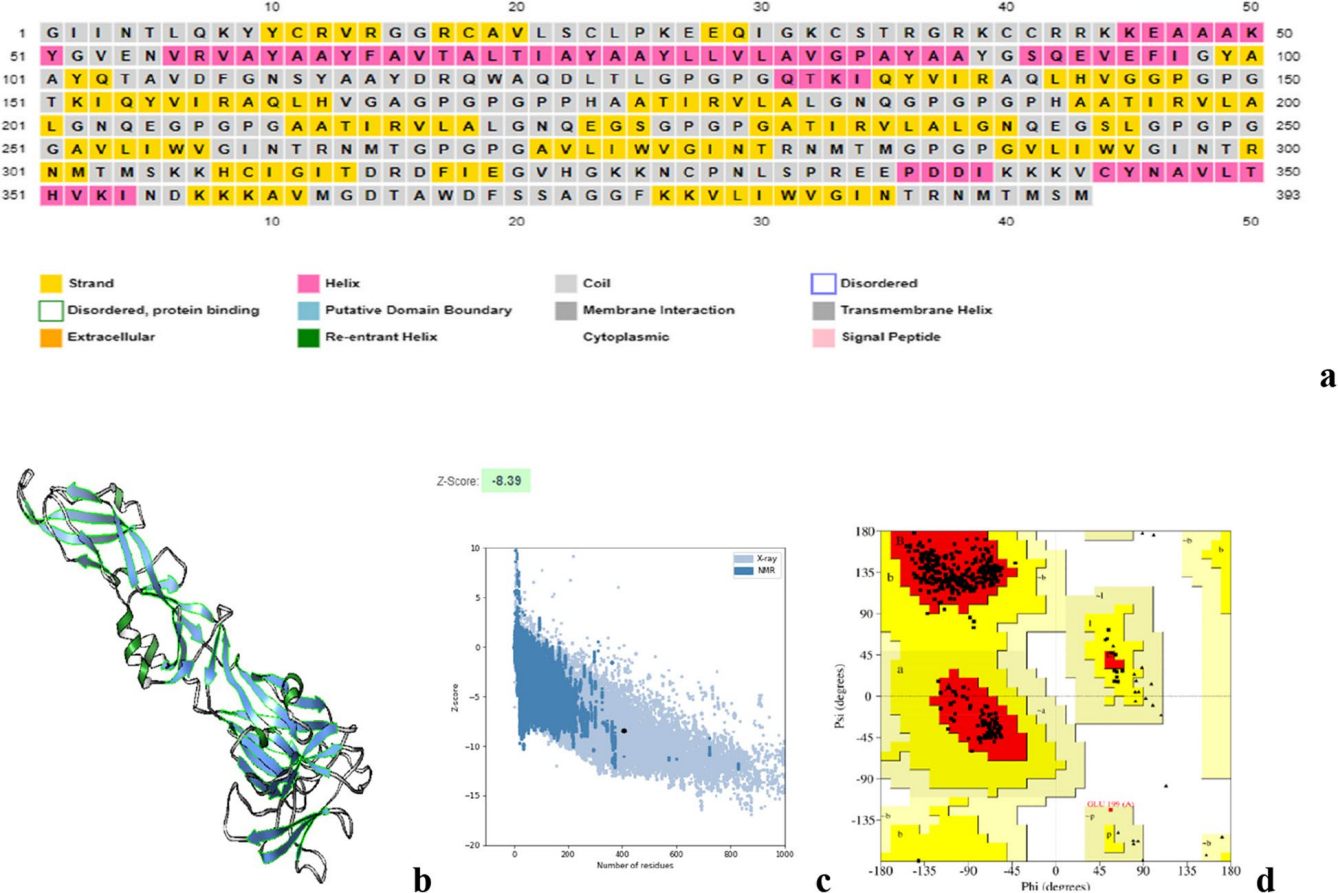
**a** The MESV construct contains α-helix (pink), beta strands (yellow), and random coil (gray). **b** 3D model of the final MESV construct. Green, blue, and gray color represent the alpha helix, beta strands, and coil regions, respectively. **c** 3D structure validation with a Z-score of − 8.39 followed by ProSA web. **d** Ramachandran plot analysis based on PROCHECK server showed 94.6% residues present in the favorable region, 5.1% residues present in the outlier region, and 0.0% residues present in the disallowed region

Proteome of *Homo sapiens* was first analyzed using Blastp, and results depicted that the MESV construct did not show similarity with any human protein (high or equal to 37%) and was found to be stable. Moreover, the antigenicity, allergenicity, and toxicity of the vaccine’s structure were examined. The antigenicity of the designed MESV was 0.74 and is highly antigenic, non-toxic, and non-allergenic. According to the physicochemical results shown by the ProtParam tool, the vaccine construct was composed of 393 residues with a molecular weight of 41,534.04 kDa and pI is 9.69. The construct was having 22 (Asp + Gly) residues with negative charges and 43 residues with positive charges (Arg + Lys), and Molar Ext. coefficient 73,340 M^−1^ cm^−1^ at 280 nm. The N-terminal of the sequence was having G (Gly) residue (Fig. 9a). In three distinct reference cells, the half-life of the vaccine construct was calculated. The vaccine construct’s mean half-life was examined to be 30 h for mammalian reticulocytes, in vitro), > 20 h for yeast (in vivo), > 10 h (*Escherichia coli*) in vivo. The instability index (II) is computed to be 17.70. This classifies the protein as stable. The aliphatic index was determined to be 85.17, while the overall average hydropathicity was calculated to be − 0.091. The 2D structure of MESV was predicted using PSIPRED. PHYRE2 (www.sbg.bio.ic.ac.uk/phyre2) is a tool for predicting the 3-dimensional structure of the MESV construct (Fig. 9b). The predicted MESV structure was refined by the Galaxy refine server. The Ramachandran plot analysis was carried out by using SAVES v6.0. To confirm the quality of the refined MESV, PROSA web server was used (Fig. 9c). Ramachandran plot was created by Saves v6.0 by applying PROCHECK. The ERRAT server was used to evaluate the calculations of the unbounded interactions in the MESV structure. Ramachandran plot analysis of the improved model showed that 94.6% of residues are present in the favored region, 5.1% residues are present in the outlier region, and 0.0% residues are present in the disallowed region (Fig. 9d). By Saves v6.0 by applying ERRAT, the refined quality score was 96.24. The z-score was − 8.39.

### Prediction of linear and discontinuous B cell epitopes of MESV

Antibodies produced by B-lymphocytes contribute to humoral immunity. The IEDB server’s ABCpred and Ellipro tools were used to predict the linear B cell epitopes of the MESV and the discontinuous B cell epitopes, respectively. A total of 29 linear epitopes were predicted by ABCpred and 2 discontinuous epitopes from the MESV’s 3D structure by DiscoTope server.

**Fig. 10:**
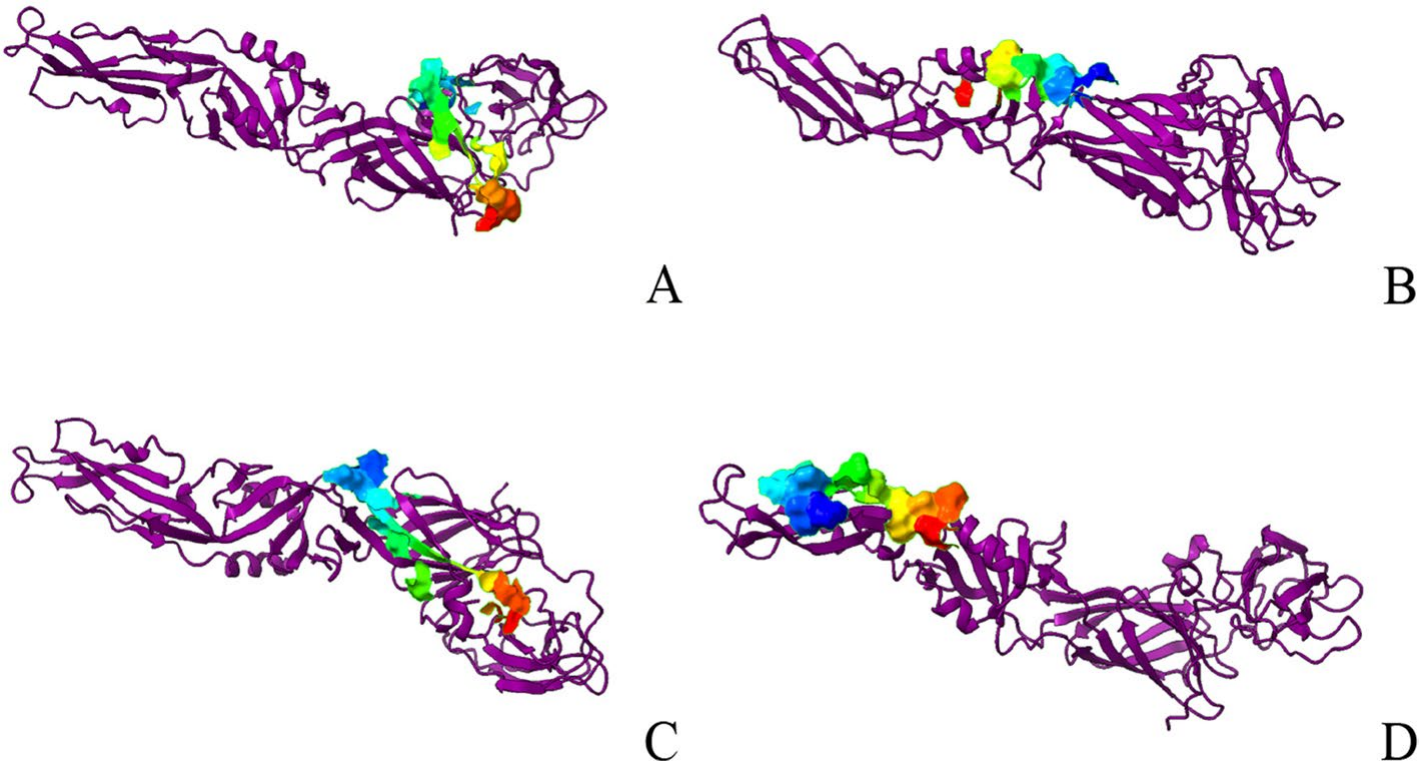
Representation of epitopes on 3D structure of MESV construct. **A** corresponds to HCIGITDRDFIEGVHG, **B** corresponds to QTAVDFGNSY, **C** corresponds QTKIQYVIRAQLHVG, **D** corresponds to PPHAATIRVLALGNQ

Using chimera, we have visualized the linear and discontinuous epitopes on the 3D structure of the vaccine construct, some are mentioned HCIGITDRDFIEGVHG (B cell), QTAVDFGNSY MHC-1 (E), QTKIQYVIRAQLHVG (MHC 2—E), PPHAATIRVLALGNQ (MHC2-E) displayed in Fig. 10.

### Analysis of molecular docking of MESV with TLR3, TLR4, and TLR8

**Fig. 11:**
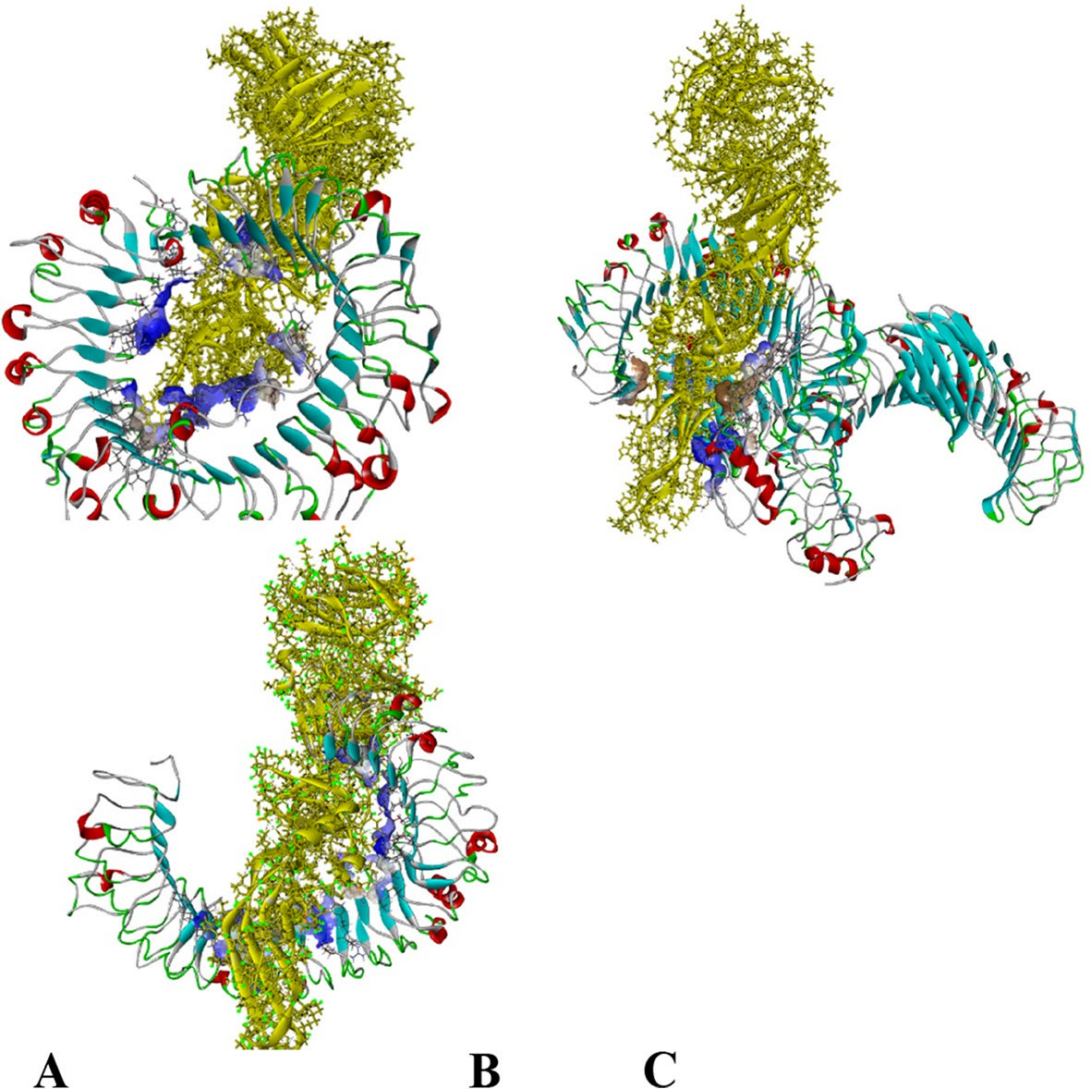
Molecular docking of TLR receptors with designed MESV along with binding interactions shown in blue shaded region visualized by Discovery Studio. **A** Docked complex of vaccine and TLR-8, **B** docked complex of vaccine and TLR-4, and **C** docked complex of vaccine and TLR-3

Molecular docking was performed to predict the binding affinities and molecular interaction pattern between the MESV construct and TLR-3, TLR-4, and TLR-8 using Maestro Schrodinger. Protein–protein interaction between the vaccine construct and TLR using Discovery Studio is shown in Fig. 11. Innate immunity relies on the interaction between immune cells TLRs and vaccines to elicit a consistent immune response. TLR can recognize the yellow fever virus structural protein efficiently resulting in the production of inflammatory cytokines.

Among the top complexes of the docking of the vaccine against TLR-3, TLR4, and TLR8 with best conformations was retrieved from Maestro Schrodinger shown in Fig. 11.

### Analysis of in-silico molecular dynamics (MD) simulation

**Fig. 12:**
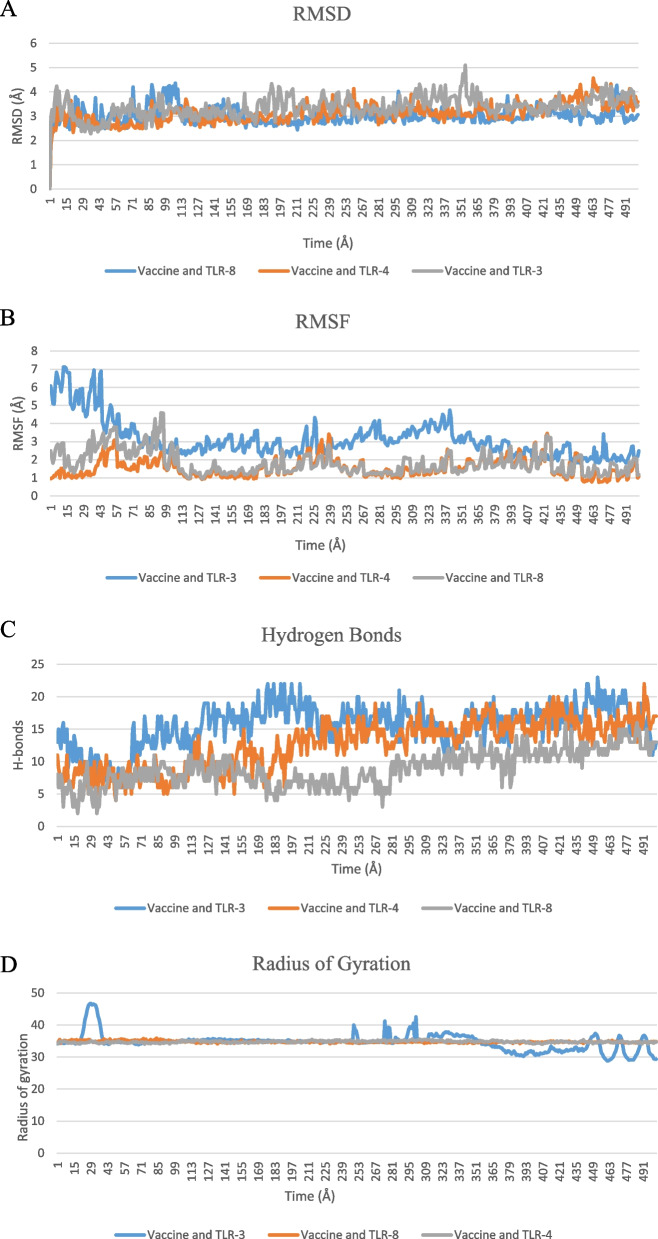
Molecular dynamics simulations. **A** RMSD, **B** RMSF, **C** hydrogen bonds, and **D** radius of gyration

The root mean square deviation (RMSD) values of c-alpha atoms of the vaccine complex were calculated for the total vaccine complexes with TLR-3, TLR4, and TLR8 receptors. The average RMSD values for the given vaccine and TLR-3 complex, vaccine, and TLR-4 complex and vaccine and TLR-8 complexes are 3.37 A, 3.96 A, and 3.02 A respectively, which demonstrates the stable nature of the vaccine complexes, shown in the given Fig. 12(A). Vaccine and TLR-3 complex showed the initial increase in RMSD descriptors up to 5.10 A at 70,799 time step whereby the upward trend stops. A relatively very less degree of fluctuation was found for vaccine and TLR complexes which indicate the structural integrity between the complexes.

The root mean square fluctuation (RMSF) values measure the average deviations of protein residues over a time period from the reference position, shown in Fig. 12(B). Thus, RMSF analyzes the complex structure’s portions that are deviating from their mean structure the most or least.

Radius of Gyration (Rg) for a given simulation trajectory gives information regarding the compressed state of the protein, where high Rg profile indicates less rigidity in the biological system. The Rg profile of the vaccine and TLR3 complex demonstrated an initial increase was observed. Therefore, the Rg descriptor graph of MESV was similar up to 245 Å, although few fluctuations were observed in both the MESV and TLR3 complexes at different timesteps. In contrast, Rg values of the TLR8 and TLR-4 complexes were similarly depicted in Fig. 12(D).

### In-silico immune simulation using C-ImmSim

**Fig. 13:**
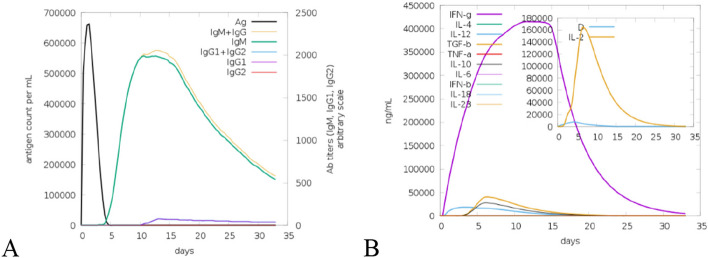
In-silico immune simulation profile of vaccine injected. **A** Antibodies. **B** Cytokines and interleukins

C-ImmSim web server was employed to perform in-silico immune simulation taking MESV as an antigen with a single time-step of injection, 10 simulation volumes, and 100 simulation steps. The primary and secondary responses were shown by predicting the activity of MESV. The host’s immune system response against the MESV as an antigen, in-silico is shown in Fig. 13. The primary immune response in the graph was characterized by high IgM and IgM + IgG concentrations, followed by IgG1 + IgG2. Therefore, the in-silico immune response graphs show the successful generation of the immune response against MESV and clearance after several encounters.

### In-silico cloning of vaccine construct with pBR322 E. coli expression system

**Fig. 14:**
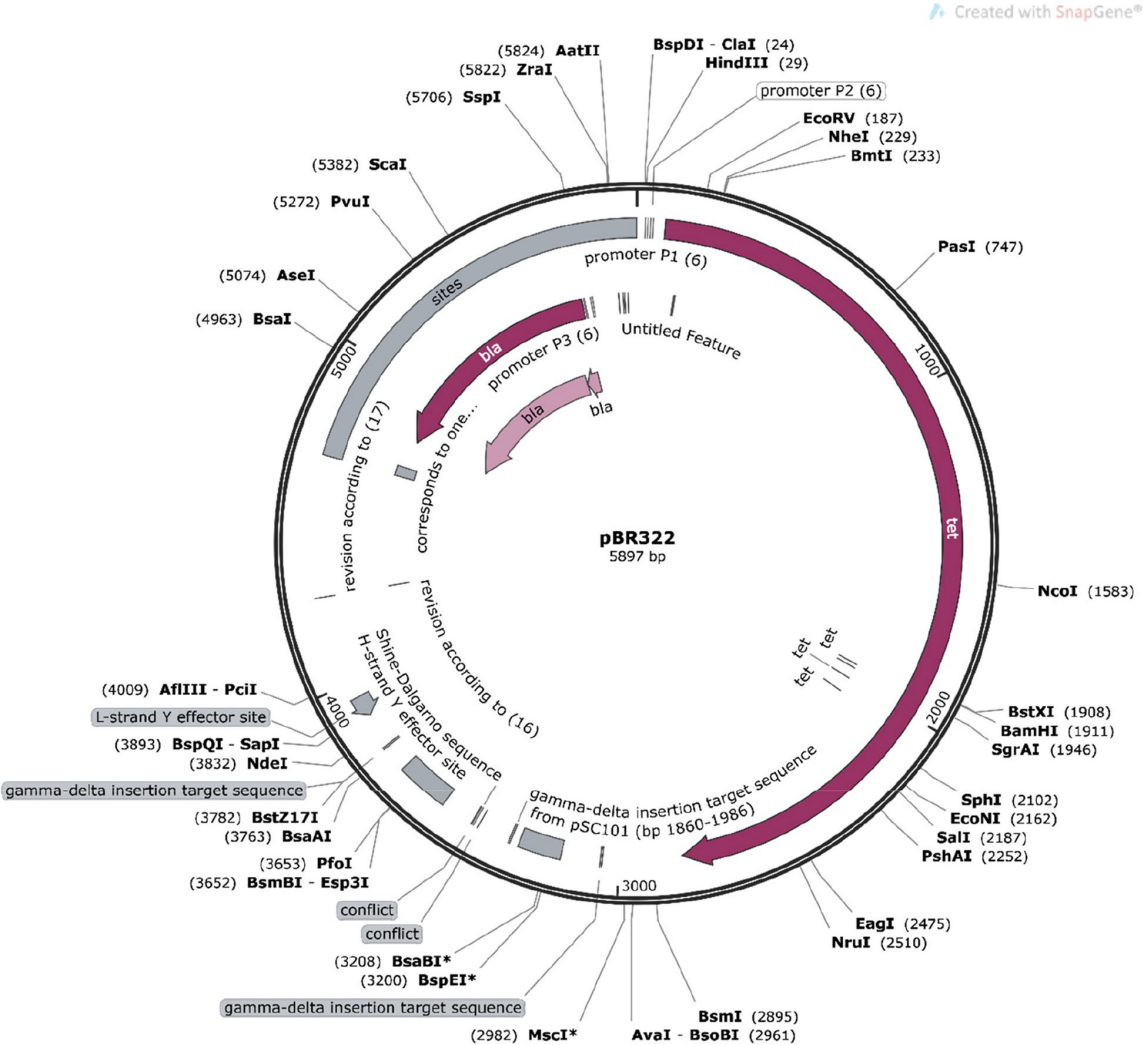
In-silico cloning

In-silico cloning of vaccine construction was performed using codon optimization shown in Fig. 14. JCat webserver was used to reverse transcribe the vaccine construct into cDNA. Multi-epitope vaccine’s cDNA derived from JCat was subjected to restriction cloning into pBR322 *E*. *coli* expression vector (4361 bps) and cloned to BamH1 site (375 bps) using Mendelgen tool. The CAI-value and GC-content score of an optimized sequence of the vaccine was predicted 1 and 55.33 resp, for 1536 nucleotides. Higher CAI value indicated high gene expression of vaccine sequence in the host cell suggesting a potential candidate to stimulate both humoral and cellular immune response effectively.

## Conclusion

Structural proteins (prM & E) derived from the Yellow fever virus were characterized for antigenic peptide epitopes and proposed a potential MESV candidate by utilizing various computational and immune informatics driven methods. The present in-silico study could save cost and time to identify and to design a safe and effective novel multi-epitope vaccine with T and B cell epitopes. Vaccines can be evaluated for their structural, antigenic, and physicochemical profile computationally. Through molecular docking and molecular dynamics simulation studies, molecular interactions and stability index of vaccine and TLR complexes were assessed followed by in-silico immune simulations and cloning indicated the activation of the host immune system and was found to be very effective in generating humoral as well as innate immune response. Therefore, the designed multi-epitope vaccine can be considered for further studies. However, to prove the efficacy of the proposed multi-epitope vaccine construct, further in-vitro and in-vivo experimental studies are required.

## Data Availability

Not applicable.
